# Consultation Intervention Rates for the Otolaryngology Service: A Large Metropolitan Hospital Experience

**DOI:** 10.51894/001c.11596

**Published:** 2020-01-30

**Authors:** Matt Mors, Colin Bohr, Michael Fozo, Carl Shermetaro

**Affiliations:** 1 Otolaryngology- Head and Neck Surgery McLaren Oakland Regional Medical Center; 2 Otolaryngology- Head and Neck Surgery St. John Hospital and Medical Center

**Keywords:** quality improvement, otolaryngology referrals, consultation, indications, otolaryngologic patient assessments

## Abstract

**CONTEXT:**

The purpose of this study was to evaluate the types of consultations received by an otolaryngology service at a 772-bed large metropolitan, MI-based hospital.

**METHODS:**

The authors performed a retrospective review of the specific types of consultations received during calendar year 2016.

**RESULTS:**

A total of 518 consultations were reviewed and analyzed by the first and second authors (MM, CB). Consultations with low intervention rates included dysphagia (difficulty swallowing) (32.3%), dysphonia (difficulty speaking) (16%), otalgia (earache) (20.8%), hearing loss (13.3%), rule out vocal cord dysfunction (0%), and vertigo/dizziness (0%). Epistaxis (nosebleed) was the most frequent reason for consultations, and angioedema (lip or airway swelling) was the most common airway-related consultation. Notably, emergent or urgent surgery was only performed on 4.6% of sample patients. Several common consultation reasons (e.g., longer-term hearing loss evaluation and cerumen (“earwax”) removal) could have been deferred for clinic-based evaluation where audiograms and microscopes are more readily available.

**CONCLUSIONS:**

These findings suggest areas for continuing education for primary care provider and resident education to place more appropriate hospital consultations. Annual resident lectures to prepare junior residents for the most common call scenarios (i.e., control epistaxis and incision and drainage of peritonsillar abscesses) could be helpful in this area. In addition, didactic lectures for primary care physicians on how to evaluate patients with dysphagia may be of value as this was a common consult for otolaryngologist referrals.

## INTRODUCTION

Hospital and emergency department (ED) consultations for otolaryngology (i.e., Ear, Nose, and Throat or “ENT”) services span a wide range of patient complaints with varying degrees of severity and urgency. Although there are ENT consults that must be addressed more urgently in both ED and hospital settings, some proportion of consults could be deferred for clinic-based evaluation and management. With rising healthcare costs, increased pressures to obtain higher patient satisfaction scores, and resident hour restrictions, the need to examine this phenomenon of the ENT service hospital consultations to increase healthcare efficiency and maintain patient safety has been emphasized.^1–3^

A literature search by the authors identified only two papers describing projects that had analyzed referrals placed to otolaryngologists in the hospital setting.^4,5^ However, consultation patterns have been studied within other specialties in order to evaluate their overall appropriateness or the appropriate treatment setting of hospital or clinic-based specialists.^6–18^

### Purpose of Study

The aim of this study was to identify trends in ED and hospital ENT consultations as it pertained to intervention rates, ENT sign off rates, and the types of intervention performed by ENT service providers. Identifying such trends could help foster possible areas of education for lesser-experienced residents and other consulting services. Additionally, examining possible instances of such trends at a larger 772-bed metropolitan hospital (i.e. especially those conditions requiring little ENT intervention) could serve to identify areas where efficiency and potentially cost savings can be increased within similar healthcare systems.

The authors’ ENT service covered four area hospitals. St. John Medical Center, a designated teaching hospital and Level II Trauma Center, was the largest of these with 772 beds. At the time of this study, the ENT service did not take facial trauma call (ENT services involving any physical trauma to the face).

## METHODS

Before data collection, institutional board review study approval was obtained. Electronic health records were then examined of all hospital consultations received by the ENT service during the time period 1/1/2016-12/31/2016. This one-year period was chosen to provide a typical representation of the variety of consults the authors’ ENT service receives. Sample patient age and gender were recorded.

Additional information gathered included: reason for ENT consultation, related diagnosis, intervention performed (if any), ENT sign off reasons, reason for recommending post-hospital clinic-based evaluation, and other ENT service recommendations. Some of these data points have been used in similar studies in the past to analyze consultation patterns.^5^ This study included all documented pediatric and adult consultations.

## RESULTS

Out of the 518 ENT consultation notes reviewed, there were 72 different consultation reasons and 110 different diagnoses. Patient demographics are listed in Table 1. Of note, the ENT service only saw a subgroup of 32 (6.2%) pediatric patients, which may be due to a pediatric otolaryngology group having covered this hospital as well. The median patient age was 56 years old (SD 20.9) with 243 (46.9%) males and 275 (53.1%) females. Patient ages ranged from 0 to 96 years including the 32 pediatric patients. (Table 1)

**Table 1.** Sample Patient Demographics

*Characteristic*	Value
	
Age (in complete years	
Mean	53.2
*Median*	56
*Age Range*	0-96
*Sex (%)*	
*Male*	243 (46.9%)
*Female*	275 (53.1%)
*Adult Patients*	486 (93.8%)
*Pediatric Patients*	32 (6.2%)

### Types of ENT Consultations

The most common consult indications and primary diagnoses are listed in Tables 2 and 3. As indicated in these tables, the documented reasons for ENT consultations did not necessarily correlate to the patient’s admitting diagnosis or chief complaint. For example, a patient may have been admitted for stroke evaluation and develops epistaxis during their hospital stay. Additionally, a consultation reason may be for a chronic issue that had been earlier brought to healthcare providers’ attention (e.g., a patient admitted for chest pain who mentions a several month history of voice changes).

As seen in Table 2, **Epistaxis** was the most common consultation reason and diagnoses. Out of 66 consultations there was an intervention rate of 57.5% (38 of 66). Of these, nasal cautery was performed in 19.6% (n = 13), and nasal gauge packing placed in 37.8% (n = 25). The bleeding had resolved at the time of ENT evaluation in the remaining 42.4% (28) patients with medication added on 23 of these. A total of 52 of 66 patients were concluded after initial evaluation.

Recommendation for clinic-based evaluation was made in epistaxis patients, and ten patients were followed while hospitalized for nasal pack management or due to other co-morbidities (e.g. laryngeal cancer, or nasogastric tube trauma). No surgeries were performed.

**Angioedema** was the most common airway related consultation making up 27.4% of our airway-related ENT consults (angioedema (lip or airway swelling), airway assessment, stridor (high-pitched wheezing), and tracheostomy issues). Out of the 25 consultations there was an intervention rate of 84% (21 of 25). Two misdiagnoses were noted: one hypopharyngeal mass and one with no issues found on examination. Of the 23 angioedema diagnoses, a medication was added or removed on 82.6% (19 of 23), an FFL (flexed fiberoptic laryngoscopy) performed on 91.3% (21 of 23) and a recommendation for intubation on 4.7% (one of 21) patients.

**Table 2.** Most Common Reasons for Consultations

*Requested Consultation*	No.	(% of total sample)
		
Epistaxis	66	(12.7%)
Dysphagia	34	(6.6%)
Dysphonia	25	(4.8%)
Angioedema	25	(4.8%)
Otalgia	24	(4.6%)
Peritonsillar abscess (PTA)	24	(4.6%)
Airway Assessment	23	(4.4%)
Throat/Oral Pain	22	(4.2%)
Stridor	21	(4.1%)
Facial Swelling	19	(3.7%)
Hearing Loss	15	(2.9%)

**Table 3.** Most Common Diagnoses

*Primary Diagnosis*	No.	(% of subgroup)
		
Epistaxis	66	(12.7)
Dysphagia	27	(5.2)
Angioedema	27	(5.2)
PTA/Phlegmon (i.e. localized area of acute inflammation)	26	(5.0)
Vocal Cord Paralysis	25	(4.8)
No Problem Documented	24	(4.6)
Dysphonia	14	(2.7)
Tonsillitis	14	(2.7)
Tracheostomy Related Issue	13	(2.5)
Otitis Media	11	(2.1)
Sinusitis	11	(2.1)

### Intervention Consultations

The most common ENT consult interventions involved adding or removing medication (n = 279, 53.9%) and flexible fiberoptic laryngoscopy (n = 177, 22.6%). One common example of a medication started was saline spray to provide improved nasal lubrication for epistaxis. The ENT service concluded a total of 290 (56%) of consults after initial evaluation. Reasons for this included: a) non-acute complaint (n = 184 of 290, 63.4%), a non-ENT related diagnosis (39, 13.4%), the problem resolving with intervention (33, 11.4%), and the problem resolving without intervention (29, 10%).

### Non-Intervention Consultations

For these analyses, a “no acute intervention” (NAI) consult was defined as when the ENT service signed off after initial evaluation and had no procedures performed. Exclusions included situations in which emergent or urgent diagnoses were made (e.g., consultations requiring immediate airway intervention).

This could also have included patient scenarios involving a non-ENT related issue, patients whose complaints resolved without intervention, and those patients recommended for discharge after initial evaluation. Intervention rates were then calculated as a percentage of intervention compared to the total number of consults for any given complaint.

**Dysphagia:** consultations had the most frequent NAI rate of 32.3% (11 of 34) (Table 4). Eleven different diagnoses were made with dysphagia (n = 27), laryngeal thrush (n = 3), supraglottic cyst (n = 2) and oral cavity mass (n = 2) being the most common. No emergent or urgent surgeries were performed and no recommendations for intensive care unit (ICU) admission were made for any sample patients. Clinic-based evaluation was recommended in 13 (48.1%) of 27 patients with a dysphagia diagnosis since their difficulty swallowing was not causing failure to thrive or associated airway symptoms.

**Dysphonia** consultations had an acute intervention rate of 16% (4 of 25). Nine different diagnoses were made with dysphonia (n = 14), and vocal cord paralysis/paresis (n = 8) being the most common. Urgent surgery was performed on one patient with acute onset hoarseness after an assault resulting in a dislocated cricoarytenoid joint. One patient was admitted to the ICU after acute onset hoarseness following their carotid endarterectomy. Post-hospital clinic evaluation was recommended in 12 of 14 patients with a dysphonia diagnosis.

**Otalgia** had an acute intervention rate of 20.8% (5 of 24). Nine different diagnoses were made with referred otalgia/TMJ dysfunction (n = 13), and otitis externa (n = 5) being the most common. Medications were added on 16 patients, and 11 recommended for post-hospital evaluation/monitoring. No surgeries were performed on this group. Of note, over half of the diagnoses are not ear related (referred otalgia/TMJ dysfunction). Those problems requiring acute intervention were infectious in nature (i.e., one case each of mastoiditis, zoster ear infection, and ear cartilage infection).

**Hearing loss** had an acute intervention rate of 13.3% (2 of 15). The two cases requiring acute intervention were diagnosed as sudden sensorineural hearing loss. Four different diagnoses were made with longstanding hearing loss (n = 8) and otitis media (n = 4) being the most common diagnoses. An audiogram was ordered on four patients, and outpatient follow-up recommended for 13 patients.

**Rule out vocal cord dysfunction** had an acute intervention rate of 0% (0 of 14). Six different diagnoses were made with COPD/asthma exacerbation (n = 3), vocal cord paralysis (n = 3), and no problem found (n = 3) being the most common. No surgical intervention or ICU admissions were indicated in these patients. Clinic-based evaluation was recommended for nine patients.

**Vertigo and dizziness** had an acute intervention rate of 0% (0 of 13). Four different diagnoses were made with unspecified vertigo/dizziness (n = 9), and BPPV (n = 2) being the most common. Medications were added onto three patients and the Epley maneuver was performed on two patients. The Epley

Maneuver is a repositioning technique clinicians and patients utilize to treat BPPV. Post-hospital evaluation was recommended in nine patients.

Consult reasons with lower intervention rates are listed below in Table 4 in order of frequency rather than intervention rate.

**Table 4.** Consultations With Lower Intervention Rates

*Consult Indication*	No.	(% of subgroup)
		
Dysphagia	11/34	(32.3)
Dysphonia	4/25	(16)
Otalgia	5/24	(20.8)
Hearing Loss	2/15	(13.3)
R/O Vocal Cord Dysfunction	0/14	(0)
Vertigo/Dizziness	0/13	(0)

### Consultation Patient Dispositions

Fifteen (71.4%) of 21 patients were admitted to the ICU. Two patients were already intubated at the time of our evaluation. Sign off after initial ENT consultation occurred on four patients with the majority being followed for improvement while inpatient.

Other specialty services were consulted in 40 (7.7%) of 518 cases with oral maxillofacial surgery (n = 8) and gastroenterology (n = 8) being the most common.

A total of 24 consultations required urgent or emergent surgery. The most common reasons for consultation were: stridor (n = 8), dyspnea (n = 3), airway assessment (n = 2) and findings on imaging (n = 2). Based on available documentation, this indicates that most urgent or emergent surgeries were the result of impending airway compromise. There were 12 different diagnoses listed in this group with the most common being laryngeal mass (n = 7), vocal cord paralysis (n = 6), and oropharyngeal mass (n = 2).

There were nine emergent surgeries performed on sample patients. They included six tracheostomies, a conversion of a cricothyrotomy to a tracheotomy, micro suspension with direct laryngoscopy and debulking of a laryngeal mass (to improve an upper airway obstruction), and balloon dilation of subglottic stenosis. The balloon dilation was performed by the Pulmonology service.

The three most common surgeries performed were micro suspension with direct laryngoscopy and debulking of laryngeal mass (n = 4), direct laryngoscopy with biopsy (n = 4), and tracheostomy (n = 3).

Intubation was recommended for four sample patients. Consultation reasons included stridor, angioedema, neck mass, and airway assessment. Diagnoses included supraglottic edema, angioedema, Ludwig’s angina (i.e., cellulitis of the floor of mouth), and vocal cord paralysis.

## DISCUSSION

There is currently little literature concerning ENT consultation patterns. There have been two studies to date that examined overall trends of consultation, ^4,5^ with another study examining ENT intervention rates for nasal bone fractures after the implementation of a treatment algorithm.^18^ This is the first apparent paper that addresses ENT intervention rates, although this phenomenon has been examined in other specialties.^14–16^ In 2017, Choi et. al., does make mention of specific consults that are deferred for post-hospital clinic evaluation without any evaluation by ENT in the hospital setting.^5^

Based on these findings, several complaints demonstrated low intervention rates. Consultations for dysphagia, dysphonia, otalgia, hearing loss, rule out vocal cord dysfunction, and vertigo/dizziness were typically referred to our ENT office for further management without acute intervention in the hospital. Out of the 125 consults for the above reasons only one urgent surgery and one recommendation for ICU placement was made. There were also two cases of sudden sensorineural hearing loss and one case of mastoiditis that required immediate treatment.

Importantly, all of these sample patient’s complaints that required acute intervention were acute in nature. The remainder of the ENT consults under study were either longstanding or did not warrant further hospital workup. This finding suggests that triaging certain consultations could be safe and lead to greater healthcare efficiency when the acuity and severity of symptoms are addressed during discussion with the ENT consulting team.

Also worth noting is the otolaryngologist’s perceived role in epistaxis. In this sample, epistaxis was the most common consult and diagnosis. However,

bleeding had resolved in 42.4% of patients at the time of our initial ENT evaluation. Anecdotally, many of these patients had no intervention by the primary team including conservative therapy (e.g., instruction to apply pressure to the nose, nasal cannula avoidance, or nasal saline use), or lacked a significant history of epistaxis at time of initial ENT examination. The low rate of nasal cautery (19.6%) would likely have been higher in clinic settings were the appropriate instrumentation is readily available.

Given these results, it may be prudent to develop consultation algorithms and provide education for hospital staff to better identify those patients requiring ENT evaluation. With the limitations to resident work hours and increased awareness of physician burnout, physician time management has become an area of increased interest.^19–21^ The average time to gather equipment, evaluate the patient, and document the ENT encounter was approximately 60 minutes according to a resident poll from our institution. This period is shorter than that reported by Lanigan et al.^18^

By contrast, physicians in our practice can typically see four to six patients per hour in clinic. This not only highlights the inefficiency of evaluating and treating many hospital patients for milder ENT symptoms, but has also been shown to represent a significant contribution to weekly ENT physician work hours.^18^ At present, our ENT service is evaluating how to best incorporate these study results into our care delivery processes.

It is difficult to obtain consistent data regarding cost to patient due to the variety of insurance companies, plans, contractual rates, secondary insurances and patient deductibles. Certainly, the additional costs of ENT specialist consultations will increase the overall expense of a patient’s hospital stay. However, further studies are needed to define the exact excess costs of unnecessary consultations.

These findings also suggest areas for potential education for the emergency department, floor staff, primary care physicians, and junior residents. Lectures and discussions aimed toward the more appropriate management of patients with ENT complaints could create a more efficient and cost-effective healthcare system, and better prepare residents to recognize and treat both common and emergent complaints.^22^ This could include resident training sessions/lectures for not only junior otolaryngology residents, but also residents from other services.

The importance of triaging cases when specialist intervention is in fact required has been clearly defined by other specialties. In 2013, Hu et al analyzed hospital dermatologic complaints and found a misdiagnosis in 45% of admissions and noted a positive impact for optimizing treatments.^6^ Similar findings have been found in a number of other studies with dermatology, vascular surgery and neurology consults.^7,8,15^ Since many of our sample ENT consultations were derived from symptoms rather than diagnoses, our analyses of misdiagnosis rates in this sample would have been difficult.

In 2013, Russell et. al., made an argument for an ENT hospitalist in order to improve resident education, patient satisfaction, better collaborative relationships, increased RVUs for surgical and bedside procedures, and improved efficiency of post-hospital clinics.^4^ Admittedly, objective measures are lacking for some of these categories. Roberts et. al. expressed a similar argument regarding Neurology service consultations.^15^

### Limitations

One limitation of this study includes our use of retrospective data. Additionally, we were unable to determine the effects of ENT consultations on patient’s length of stay or the direct patient costs, and hospital and emergency room ENT consultations could not be reliably separated. The trends seen at our institution may not necessary reflect those of other facilities, though previous studies have shown similar results.^2^

## CONCLUSIONS

Based on the findings of this study, there are opportunities to improve the quality of many ENT hospital consultations. In this study, several common types of ENT consultations could more appropriately be deferred for clinic-based evaluation without challenges to patient safety. The economic effect of triaging consults for clinic-based evaluation certainly needs to be more rigorously studied. Although consultation patterns will vary from institution to institution, examining these types of consultation trends will also provide areas for future quality improvement studies.

### Conflict of Interest

The authors declare no conflict of interest.

## References

[ref-11345] Bodenheimer T. (2005). High and Rising Health Care Costs. Part 1: Seeking an explanation. Ann Intern Med.

[ref-11346] Chow A., Mayer E., Darzi A., Athanasiou T. (2019). Patient-reported outcome measures: The importance of patient satisfaction in surgery. Surgery.

[ref-11347] Bilimoria K.Y., Hoyt D.B., Lewis F. (2017). Perspective of the FIRST Trial investigators on Accreditation Council for Graduate Medical Education changes in resident work environment and duty hours. JAMA Surg.

[ref-11348] Russell M.S., Eisele D., Murr A. (2013). The otolaryngology hospitalist. Laryngoscope.

[ref-11349] Choi K.J., Kahmke R.R., Crowson M.G., Puscas L., Scher R.L., Cohen S.M. (2017). Trends in Otolaryngology consultation patterns at an academic quaternary care center. JAMA Otolaryngology-Head & Neck Surgery.

[ref-11350] Hu L., Haynes H., Ferrazza D., Kupper T., Qureshi A. (2013). Impact of specialist consultations on inpatient admissions for dermatology-specific and related DRGs. J Gen Intern Med. J Gen Intern Med.

[ref-11351] Bauer J., Maroon M. (2010). Dermatology inpatient consultations: A retrospective study. J Amer Acad of Dermatol.

[ref-11352] Koh C., Walker S. (2007). Vascular Surgery Consults: A significant workload. ANZ J Surg.

[ref-11353] O’Malley N.T., O’Daly B., Harty J.A., Quinlan W. (2011). Inpatient consultations to an orthopaedic service: The hidden workload. Irish J Med Sci.

[ref-11354] Sullivan J., Forde J., Creagh T.. (2013). A review of inpatient urology consultations in an Irish tertiary referral centre. Surg-J Royal Coll Surgeons Edinburgh Ireland.

[ref-11355] Garcia-Ramos R., Morales I., Vela A.. (2009). Analysis of hospital consultations to Neurology in a tertiary hospital. Neurolog.

[ref-11356] Donohoe M.T., Kravitz R.L., Wheeler D.B., Chandra R., Chen A., Humphries N. (1999). Reasons for outpatient referrals from generalists to specialists. J Gen Intern Med.

[ref-11357] Kohlert S., Murphy P., Tse D., Liddy C., Afkham A., Keely E. (2018). Improving access to otolaryngology-head and neck surgery expert advice through eConsultations. Laryngoscope.

[ref-11358] Lewis P.R., Dunne C.E., Wallace J.D.. (2017). Routine neurosurgical consultation is not necessary in mild blunt traumatic brain injury. J Trauma Acute Care Surg.

[ref-11359] Roberts K., Costelloe D., Hutchinson M., Tubridy N. (2007). What difference does a neurologist make in a general hospital? Estimating the impact of neurology consultations on in-patient care. Irish J Med Sci.

[ref-11360] Joseph B., Aziz H., Sadoun M.. (2013). The acute care surgery model: Managing traumatic brain injury without an inpatient neurosurgical consultation. J Trauma Acute Care Surg.

[ref-11361] Joseph B., Aziz H., Pandit V.. (2014). Prospective validation of the brain injury guidelines: Managing traumatic brain injury without neurosurgical consultation. J Trauma Acute Care Surg.

[ref-11362] Lanigan A., Lospinoso J., Bowe S.N., Laury A.M. (2017). The Nasal Fracture Algorithm: A case for protocol-driven management to optimize care and resident work hours. Otolaryngology-Head Neck Surg.

[ref-11363] Fletcher A.M., Pagedar N., Richard Smith (2012). Factors correlating with burnout in practicing otolaryngologists. Otolaryngology-Head Neck Surg.

[ref-11364] Curtis S.H., Miller R.H., Weng C., Gurgel R.K. (2014). The effect of duty hour regulation on resident surgical case volume in otolaryngology. Otolaryngology-Head Neck Surg.

[ref-11365] Reiter E.R., Wong D.R. (2005). Impact of duty hour limits on resident training in otolaryngology. Laryngoscope.

[ref-11366] Chin Christopher J., Roth Kathryn, Rotenberg Brian W., Fung Kevin (2014). Emergencies in otolaryngology-head and neck surgery bootcamp: A novel Canadian experience. The Laryngoscope.

